# A Bioinformatics-Based Study on Methylation Alterations of the FBLN1 Gene in Hippocampal Tissue of Alzheimer’s Disease Model DKO and DTG Mice

**DOI:** 10.3390/ijms25169036

**Published:** 2024-08-20

**Authors:** Rui Tang, Yue Jian, Zhimin Hu, Li Li, Haitao Wang, Pengyu Miao, Zhihui Yang, Mingxi Tang

**Affiliations:** 1 School of Basic Medical Sciences, Southwest Medical University, Luzhou 646000, China; 20210199120038@stu.swmu.edu.cn (R.T.);; 2 The School of Clinical Medical Sciences, Southwest Medical University, Luzhou 646000, China

**Keywords:** DNA methylation, *FBLN1* gene, single-cell sequencing, Alzheimer’s disease, DKO mice, DTG mice

## Abstract

Alzheimer’s disease (AD) is characterized by progressive cognitive decline and late-stage neurobehavioral issues marked by amyloid-beta plaques and Tau protein tangles. This study aims to investigate Fibulin-1(FBLN1) gene expression in the hippocampal tissue of *Presenilin-1/Presenilin-2* conditional double-knockout (DKO) and double-transgenic (DTG) mice, using single-cell sequencing and experimental methods to verify abnormal methylation status and correlation with AD. Genomic DNA from DKO and DTG mice was used for genotyping. Reduced Representation Bisulfite Sequencing (RRBS) identified 10 genes with abnormal methylation changes, with protein–protein interaction (PPI) analysis highlighting five core genes, including *FBLN1*. Single-cell sequencing, RT-PCR, and Western blotting (WB) were used to analyze *FBLN1* mRNA and protein levels in the hippocampal tissues of early-stage and mid-stage AD DKO, DTG, and CBAC57 mice. RRBS identified 10 genes with abnormal methylation, with PPI highlighting five core genes. Single-cell sequencing showed significant *FBLN1* expression in AD groups. RT-PCR and WB indicated elevated *FBLN1* mRNA and protein levels in mid-stage AD DKO and DTG mice compared to CBAC57 mice, with no differences in early-stage AD DKO and CBAC57 mice. RRBS revealed hypomethylation of the *FBLN1* gene in mid-stage AD DKO mice. Elevated *FBLN1* expression in AD models suggests an age-dependent neurodegenerative mechanism independent of amyloid-beta deposition. This study enhances our understanding of AD’s epigenetic mechanisms, which will aid targeted diagnosis, treatment, and prognosis.

## 1. Introduction

Neurodegenerative diseases, characterized by age-dependent, irreversible, and progressive deterioration, are leading contributors to morbidity and disability among the elderly [1]. These diseases involve the loss of neurons or myelin structures, leading to central or peripheral nervous system dysfunction [1]. This process impairs motor and cognitive functions, progressively worsening with age and ultimately leading to disability and death. Alzheimer’s disease (AD) is the most prevalent neurodegenerative disorder, surpassing the combined prevalence of all other such diseases. AD primarily manifests as a central neurodegenerative disorder, characterized by progressive declines in learning, memory, and cognitive function, along with late-stage neurobehavioral abnormalities [2]. Pathologically, AD is defined by the extensive deposition of amyloid-beta (Aβ) proteins in the cerebral cortex and hippocampus, forming senile plaques (SPs), and the hyperphosphorylation of Tau proteins, resulting in neurofibrillary tangles (NFTs) [3,4]. Despite extensive research, the etiology and pathogenesis of AD remain incompletely understood. In recent years, epigenetics has emerged as a critical field of study, offering deeper insights into diseases such as AD. Epigenetic modifications, particularly DNA methylation, are closely linked to the pathogenesis and progression of AD, playing crucial roles in gene expression and regulation [5].

Studies have demonstrated that the Presenilin-1/Presenilin-2 conditional double-knockout (DKO) mouse model, created through gene-knockout technology, can replicate the pathological changes observed in clinical AD patients over time. These changes include neuronal loss, molecular alterations, electrophysiological characteristics, tissue morphology, inflammatory responses, and cognitive and emotional behaviors, even in the absence of Aβ protein deposition. This model serves as a valuable research tool for studying AD mechanisms independent of Aβ protein deposition [6,7,8]. Another extensively used AD animal model is the APP/PS1 double-transgenic (DTG) mouse. This model produces significant amounts of Aβ at an early stage, leading to its deposition in the brain and subsequent formation of senile plaques. Due to these pathophysiological characteristics, the DTG mouse model is deemed an ideal experimental model for AD research [9,10].

Therefore, to more accurately model the pathophysiological progression of human AD and obtain precise experimental results and conclusions, this study utilizes both DKO and DTG mouse models. DKO mice exhibit significant age-dependent neurodegenerative changes, while the DTG mice compensate for the lack of Aβ deposition and senile plaque formation in the DKO model, thus encompassing key pathological features of AD. With the advent and application of high-throughput sequencing technologies, Reduced Representation Bisulfite Sequencing (RRBS) has become widely used for DNA methylation detection due to its accuracy, efficiency, and cost-effectiveness [11]. Additionally, the rapid advancement of bioinformatics over the past decade has enabled comprehensive analyses of gene variations and co-expression, providing valuable insights into how these factors influence protein function and disease progression. Techniques such as molecular biology-enrichment analysis, based on molecular functions, biological processes, and cellular components, have become indispensable tools for further research.

In this study, we found abnormal methylation genes associated with AD and further analyzed the correlation between an abnormal methylation status of the *FBLN1* gene and the pathological progression of AD. This study aims to provide a basis for fundamental research on non-Aβ mechanisms in AD, as well as its prevention, diagnosis, and targeted treatment.

## 2. Results

### 2.1. Genotype Identification of Offspring in AD Model DKO and DTG Mice

**Figure 1:**
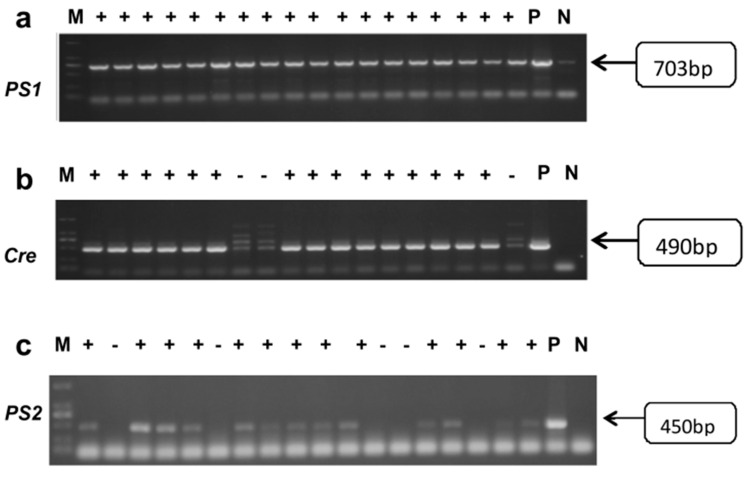
Graphs of agarose gel electrophoresis results of DNA PCR products from DKO offspring mice. (**a**) *PS1* gene results, band position 703 bp. (**b**) *Cre* gene results, band position 490 bp. (**c**) *PS2* gene result map, band position 450 bp. M: DNA Marker; P: Positive control; N: Negative control.

**Figure 2:**
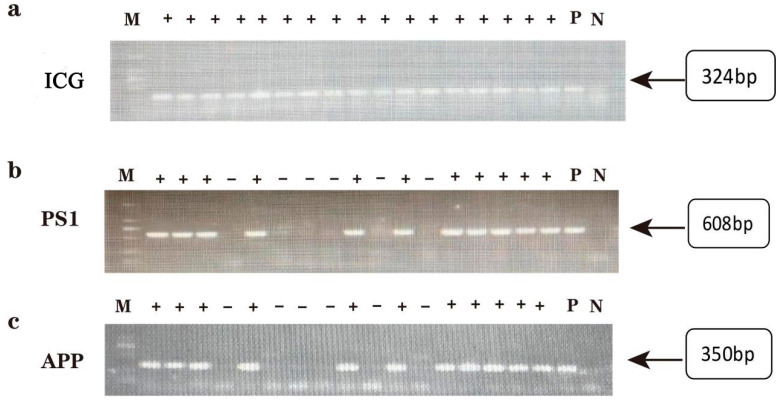
Graph of agarose gel electrophoresis results of DNA PCR products from DTG offspring mice. (**a**) Result graph of internal reference gene, band position 324 bp. (**b**) *PS1* gene results, band position 608 bp. (**c**) *APP* gene result map, band position 350 bp. M: DNA Marker; P: Positive control; N: Negative control.

The OD260/280 values of all sample DNA were not less than 1.8, and the average DNA concentration exceeded 100 ng/μL, indicating that the quality of all sample DNA met the experimental requirements. The results of agarose gel electrophoresis are presented below. The electrophoretic bands were clear and complete, with all positive results aligning with the positive controls and the expected gene-fragment sizes. The PCR amplification products of DKO mouse tail DNA displayed electrophoretic bands corresponding to the Cre, PS1, and PS2 genes, located at 490 bp, 703 bp, and 450 bp, respectively, as shown in Figure 1. Similarly, the PCR amplification products of DTG mouse tail DNA exhibited electrophoretic bands for the internal control, PS1, and APP genes, located at 324 bp, 608 bp, and 350 bp, respectively, as depicted in Figure 2. We selected only mice that simultaneously express Cre, PS1, and PS2 genes for subsequent experiments. Mice expressing any one of these genes in a negative manner were excluded from our study.

### 2.2. Methylation Screening Results of RRBS Sequencing in Hippocampal Tissue of DKO Mice

The genomic DNA results from hippocampal tissue samples of mice, detected by UV spectrophotometry, revealed OD260/280 values ranging from 1.8 to 2.0 and DNA concentrations between 102.0 and 172.0 ng/μL. These values confirm that the genomic DNA quality from mouse hippocampal tissue samples met the necessary requirements for subsequent experiments. Agarose gel electrophoresis results for each DNA sample showed clear and intact DNA bands near the wells on the agarose gel, with minimal smearing or tailing, indicating that the DNA quality was suitable for the RRBS experiments. The RRBS sequencing data were analyzed using software such as Bismark (v0.7.11) and compared to the mouse reference genome sequence, resulting in the identification of several abnormally methylated genes. As described in previous studies [12], 1338 abnormally methylated sites involving 873 genes were identified on different chromosomes during the early stages of Alzheimer’s disease (AD). The analysis of differentially methylated regions (DMRs) in functional gene areas resulted in the identification of 30 abnormally methylated genes. During the middle stages of AD, 3025 abnormally methylated sites involving 2758 genes were identified on different chromosomes (partial results are shown in Table S2). The analysis of DMRs in functional gene areas revealed 127 abnormally methylated genes, of which 95 genes exhibited increased methylation levels and 32 genes exhibited decreased methylation levels. We included the top 25 genes with more significant *p*-value expression for further analysis (results are shown in Table S3).

### 2.3. PPI Analysis

**Figure 3:**
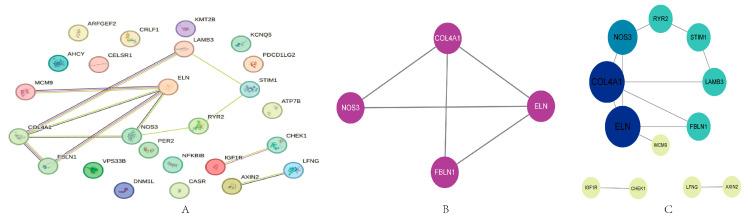
Protein–protein interaction network. (**A**) PPI of candidate genes (**B**) classification of PPI network group. (**C**) Interaction network of proteins. Nodes represent proteins and edges represent interaction relationships between proteins. Blue nodes represent a high degree of proteins; the node size is proportional to the degree of the protein in the network.

A preliminary PPI network was established based on 25 candidate genes. There are 25 nodes and 12 edges in Figure 3, with a local clustering coefficient of 0.28 (Figure 3A). MCODE was used to identify highly interconnected subnetworks, and the MCODE network analysis reveals one cluster containing four nodes (*COL4A1*, *ELN*, *NOS3*, and *FBLN1*) and five edges (Figure 3B). Four proteins in the subnetwork are COL4A1, ELN, NOS3, and FBLN1. Data for the subnetworks are shown in Table S4. To include more proteins for the study, we included the original proteins based on the subnetwork for further analysis, though the cytoHubba was used to assess the importance of the nodes in the network, quantitatively ranking the hub genes. The size of the node represents the degree value of the target; the larger the node, the greater the degree value. Nodes represent proteins and edges represent interaction relationships between proteins. Proteins with large degrees play a crucial role in the whole network. The top five proteins with degree intelligence are COL4A1, ELN, NOS3, FBLN1, and LAMB3 (Figure 3C).

### 2.4. Single-Cell RNA Sequencing Analysis of Core Genes

**Figure 4:**
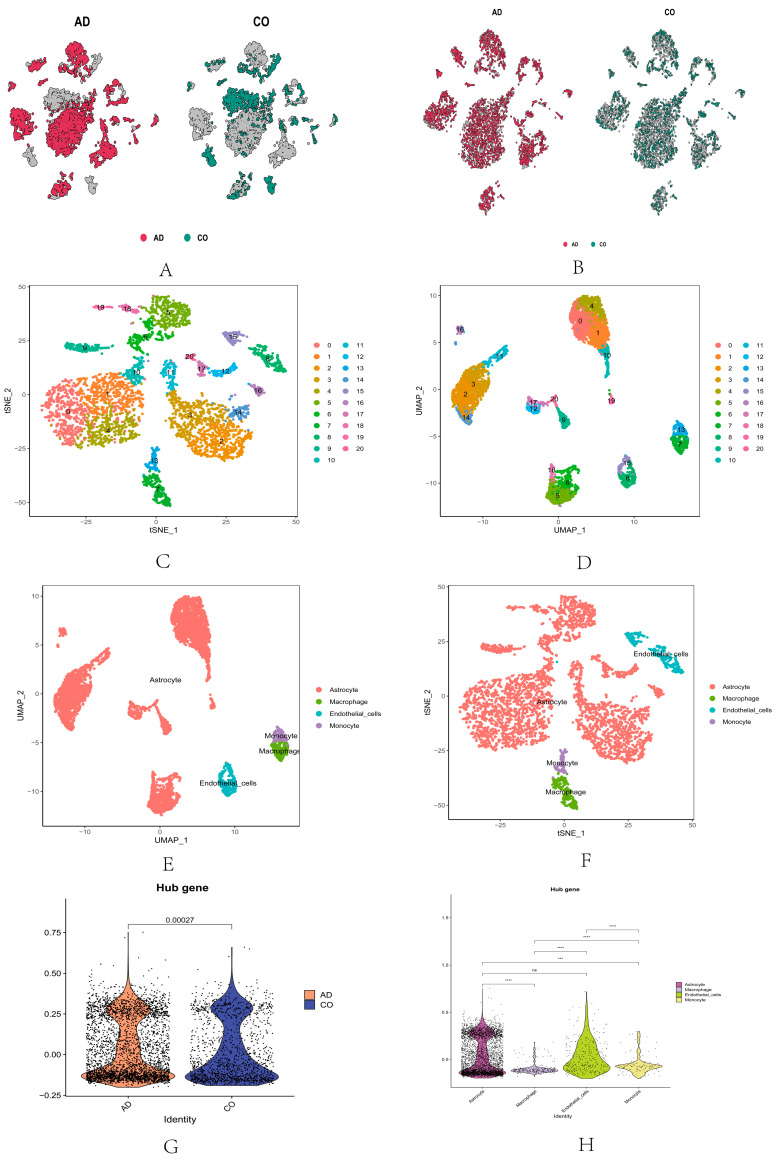
Discovery of quality control and cellular annotations in scRNA-seq data. (**A**) Cellular distribution of unfiltered AD and CO. (**B**) Cellular distribution of filtered AD and CO. (**C**) Cell clustering classification under the T-SNE algorithm. (**D**) Cell-clustering classification under the UAMP algorithm. (**E**) Cellular annotations under the T-SNE algorithm. (**F**) Cellular annotations under the UAMP algorithm. (**G**) Expression of hub genes in the AD and control group. (**H**) Expression of hub genes in cellular annotations. (ns: *p* > 0.05; *** *p* <0.001, **** *p* < 0.0001).

To further elucidate the specific roles of marker genes in AD progression, we downloaded dataset GSE161045 and performed single-cell RNA sequencing (scRNA-seq) analysis on these samples. The cells from AD and normal tissues are depicted in Figure 4A. After quality-control filtering (Figure 4B), cells from both AD and normal tissues were merged, clustered, and annotated. We identified 20 distinct cell clusters. The visualization of these clusters was achieved using the UMAP and t-SNE methods (Figure 4C,D). Each cluster’s cell identity was annotated using reference datasets from the human primary cell atlas. These clusters exhibited distinct gene-expression profiles, and marker genes specific to cell types were assigned to clusters based on their specificity.

**Figure 5:**
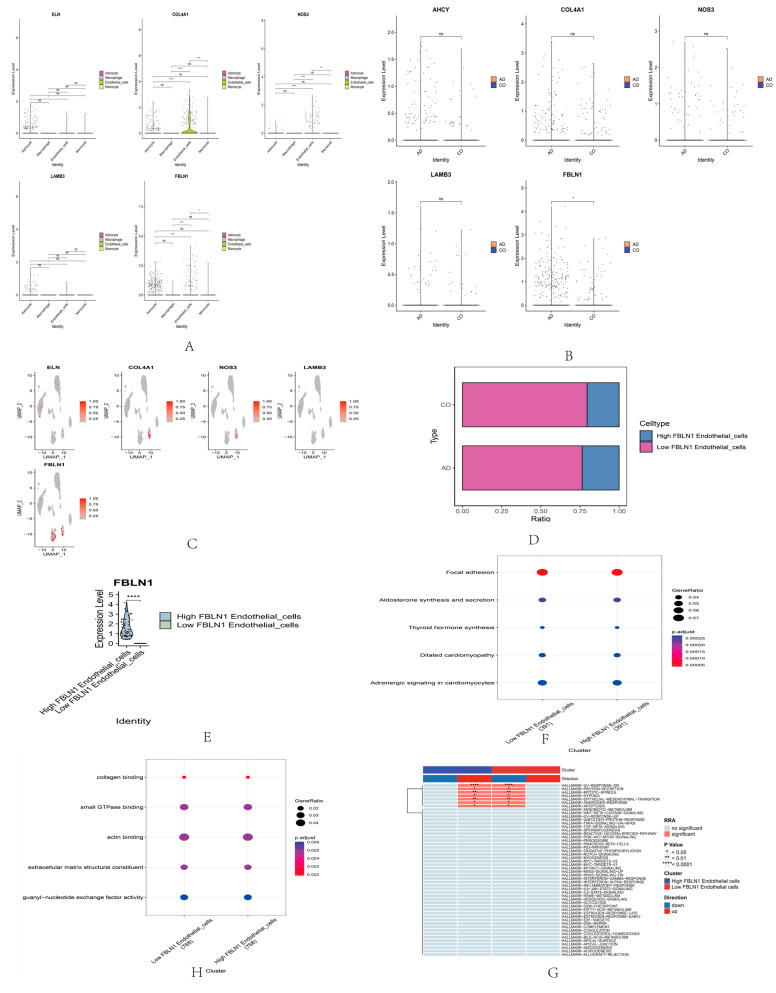
Expression and pathway analysis of *FBLN1* in endothelial cells. (**A**) Differential expression of hub genes in cell classification. (**B**) Differential expression of hub genes in AD and CO. (**C**) Expression of core genes in cellular categorization. (**D**) Expression of *FBLN1* in AD and CO in endothelial cells under the UMAP algorithm. (**E**) *FBLN1* expression in endothelial cells. (**F**) GO analysis. (**G**) KEGG analysis. (**H**) Hallmark analysis. (ns: *p* > 0.05; * *p* < 0.05; ** *p* < 0.01; *** *p* < 0.001, **** *p* < 0.0001).

An analysis of these core genes resulted in a two-dimensional plot showing the dimensional reduction of clusters, including endothelial cells, astrocytes, macrophages, and monocytes (Figure 4E,F). Then, a differential expression map of hub genes in AD and control groups (CO) was drawn (Figure 4G), indicating the difference between AD and CO (*p* < 0.05) and the differential expression map between cell clusters (Figure 4H). The differential expression of each gene in the hub between cell clusters (Figure 5A), and the differential expression of these genes in AD and control groups (CO) (Figure 5B), showed that only *FBLN1* genes differed in the AD and control groups (*p* < 0.05). The distribution and proportion of hub genes within cells are also shown (Figure 5C). *FBLN1* was found to be significantly distributed within cells, particularly in endothelial cells (*p* < 0.05). Comparing the *FBLN1* expression between the AD and control groups, we observed that *FBLN1* expression was higher in AD. The expression levels of high and low *FBLN1* expression in endothelial cells were significantly different (*p* < 0.001) (Figure 5E).

Enrichment analysis revealed that *FBLN1* is primarily associated with focal adhesion, aldosterone synthesis and secretion, thyroid hormone synthesis, dilated cardiomyopathy, and adrenergic signaling in cardiomyocytes, as shown in the GO analysis (Figure 5F).

In the KEGG analysis, FBLN1 was found to be primarily associated with collagen binding, small GTPase binding, actin binding, extracellular matrix structural constituent, and guanyl-nucleotide exchange factor activity (Figure 5G). The hallmark analysis revealed that *FBLN1* is mainly related to UV-response-dn, protein secretion, mitotic spindle, hypoxia, epithelial–mesenchymal transition, androgen response, apoptosis, and xenobiotic metabolism (Figure 5H).

### 2.5. Methylation Screening Results of FBLN1 Gene in Hippocampal Tissue of DKO Mice Based on RRBS Sequencing Data

The *FBLN1* gene is located on Chromosome 15, with abnormally methylated fragments identified in its exon region, specifically from Position 85,244,183 to 85,244,307. The methylation status of the *FBLN1* gene in the hippocampal tissue of 12-month-old female DKO mice showed significant downregulation compared to age- and sex-matched CBAC57 mice (*p* < 0.01). In contrast, although the *FBLN1* gene in the hippocampal tissue of 7-month-old female DKO mice also exhibited downregulation, this difference was not statistically significant compared to age- and sex-matched CBAC57 mice (*p* > 0.05), as shown in Table S5. These findings suggest that abnormal methylation of the *FBLN1* gene predominantly occurs during the mid-stage of AD progression and plays a crucial role in the development of AD during this period.

### 2.6. Transcriptional Expression of FBLN1 Gene in Hippocampal Tissue of DKO and DTG Mice

**Figure 6:**
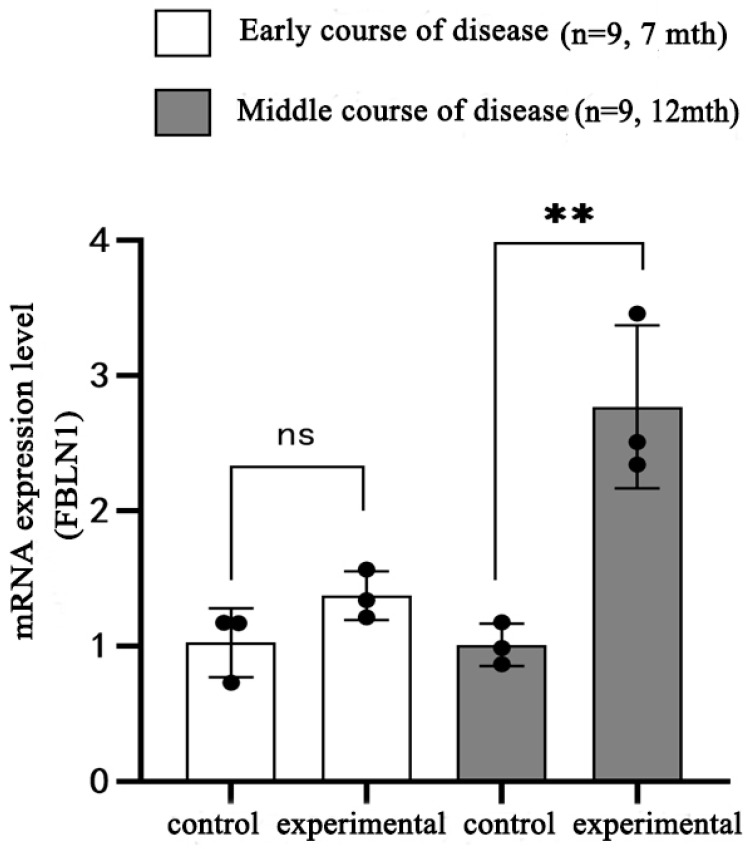
mRNA expression of *FBLN1* gene in hippocampal tissue of DKO mice with early and intermediate AD disease (ns: *p* > 0.05; ** *p* < 0.01).

**Figure 7:**
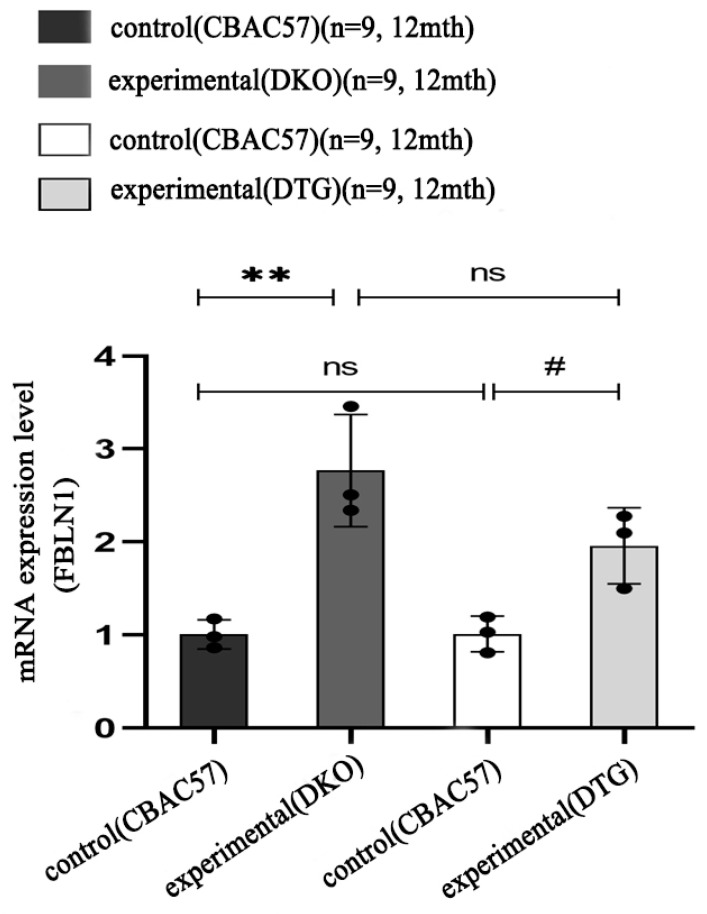
mRNA expression of *FBLN1* gene in hippocampal tissue of 12-month-old DTG mice and DKO mice (ns: *p* > 0.05; ** *p* < 0.01; # *p* < 0.05).

We first examined the mRNA expression of the *FBLN1* gene in the hippocampal tissue of 7-month-old early-stage AD model DKO mice. RT-PCR results showed no statistically significant difference in mRNA expression of the *FBLN1* gene in the hippocampal tissue of 7-month-old female DKO mice compared to same-age and same-sex CBAC57 mice (*p* > 0.05), as shown in Figure 6 and Table S6. During the mid-stage of AD progression, RT-PCR data indicated that the mRNA expression of the *FBLN1* gene in the hippocampal tissue of 12-month-old female DKO mice was significantly higher compared to same-age and same-sex CBAC57 mice (*p* < 0.01), as shown in Figure 7. Similarly, in the hippocampal tissue of 12-month-old female DTG mice, the mRNA expression of the *FBLN1* gene was also significantly increased compared to same-age and same-sex CBAC57 mice (*p* < 0.05), as shown in Figure 7.

Since DKO mice exhibit significant age-dependent neurodegenerative changes without Aβ deposition and formation of senile plaques, whereas DTG mice show significant Aβ deposition and senile plaque formation at an early stage, we investigated the relationship between the differential expression of the *FBLN1* gene at the transcriptional level and Aβ deposition. We compared the transcriptional expression of the *FBLN1* gene in the hippocampal tissue of 12-month-old female DKO and DTG mice. The results indicated that there was no statistically significant difference in the mRNA expression of the *FBLN1* gene between the 12-month-old DKO mice and same-age DTG mice (*p* > 0.05), as shown in Figure 7 and Table S6. Further analysis confirmed that there was no significant difference in mRNA expression of the *FBLN1* gene between the hippocampal tissues of DKO and DTG mice (*p* > 0.05), as shown in Figure 7 and Table S6.

### 2.7. Translational Expression of FBLN1 Gene in Hippocampal Tissue of DKO and DTG Mice

**Figure 8:**
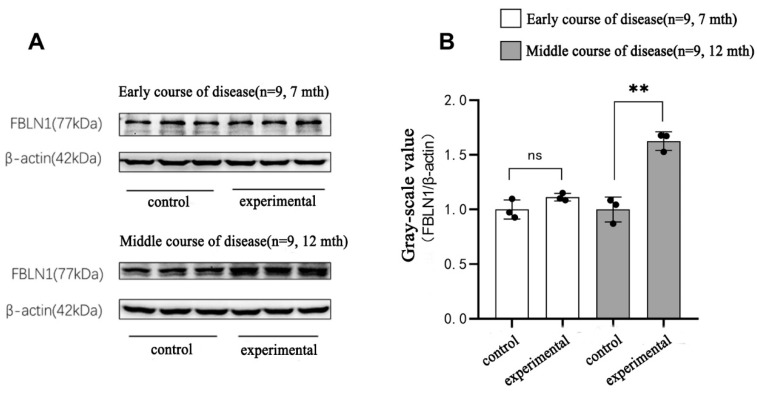
Translation level expression of *FBLN1* gene in hippocampal tissue of DKO mice with early and intermediate AD disease course. (**A**) WB bands of *FBLN1* protein expression. (**B**) Grey-scale value analysis of *FBLN1* protein (ns: *p* > 0.05; ** *p* < 0.01).

In the early stage of AD progression, Western blot (WB) data showed no statistically significant difference in the protein expression of the *FBLN1* gene in the hippocampal tissue of 7-month-old female DKO mice compared to same-age and same-sex CBAC57 mice (*p* > 0.05), as shown in Figure 8A and Table S7.

**Figure 9:**
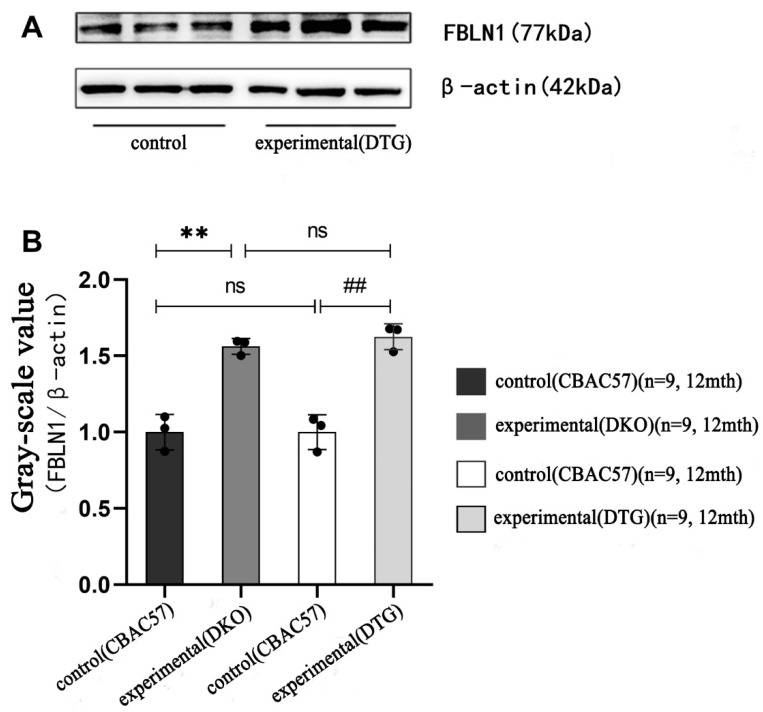
Expression of *FBLN1* gene in hippocampal tissues of DTG mice at translation level. (**A**) WB bands of protein expression of *FBLN1* gene in hippocampal tissues of 12-month-old DTG mice. (**B**) Grey-scale value analysis of FBLN1 protein (ns: *p* > 0.05; ** *p* < 0.01; ## *p* < 0.01).

In the mid-stage of AD progression, the protein expression of the *FBLN1* gene in the hippocampal tissue of 12-month-old female DKO mice was significantly higher than that in same-age and same-sex CBAC57 mice, with a statistically significant difference (*p* < 0.01), as shown in Figure 8B and Table S7. Additionally, the protein expression of the *FBLN1* gene in the hippocampal tissue of 12-month-old female DTG mice was significantly elevated compared to same-age and same-sex CBAC57 mice (*p* < 0.01), as shown in Figure 9A and Table S7.

Comparative analysis of FBLN1 protein expression in the hippocampal tissue of 12-month-old female DKO and DTG mice revealed no statistically significant difference in FBLN1 protein expression between the two groups (*p* > 0.05), as shown in Figure 9B and Table S7.

## 3. Discussion

The pathogenesis of Alzheimer’s Disease (AD) remains incompletely understood, but there are several hypotheses, including the Aβ cascade hypothesis, tau protein hyperphosphorylation hypothesis, and cholinergic hypothesis. The Aβ cascade hypothesis posits that an imbalance between the production and clearance of Aβ leads to its accumulation in the brain, forming amyloid plaques that cause neuronal degeneration and inflammatory responses, ultimately leading to cognitive impairment [13,14,15]. The tau protein hyperphosphorylation hypothesis suggests that the excessive or abnormal phosphorylation of tau protein results in its loss of the microtubule-stabilizing function, forming neurofibrillary tangles, disrupting neuronal structure and function, and causing neuronal death [16,17]. The cholinergic hypothesis proposes that damage to cholinergic neurons in AD patients leads to a decrease in acetylcholine levels, affecting memory and learning [18,19].

In this study, we utilized RRBS sequencing, single-cell RNA sequencing, RT-PCR, and Western blotting to analyze DNA methylation and gene expression in the hippocampal tissue of DKO and DTG mice. We identified several abnormally methylated genes associated with AD, with *FBLN1* showing significant differences. We further validated the methylation status and protein level changes of the *FBLN1* gene, as well as its role in different cell types and functional pathways, providing clues to its mechanism in AD pathology.

Firstly, we constructed a protein–protein interaction (PPI) network to explore the relationships and significance of the 25 abnormally methylated genes we identified. The top five proteins with degree intelligence are COL4A1, ELN, NOS3, FBLN1, and LAMB3. These genes are primarily involved in the structure and function of the extracellular matrix (ECM), as well as the regulation of cell adhesion and migration [20,21,22]. This is consistent with previous studies, indicating that ECM alterations play a crucial role in AD development and progression [23,24,25]. ECM abnormalities not only affect the morphology and function of neurons but also influence interactions between neurons and other cells such as astrocytes, pericytes, and endothelial cells, leading to neuroinflammation, blood–brain barrier disruption, and neurotrophic deficiency [25,26]. Therefore, we speculate that the abnormally methylated genes we identified may participate in AD pathology by affecting ECM composition and function.

Secondly, we utilized single-cell RNA sequencing to perform an in-depth analysis of cells from AD and normal tissues, revealing the heterogeneity and specificity of different cell types. We found significant differences in cell distribution and phenotype between AD and normal tissues, indicating that AD development involves changes in multiple cell types. We plot the differential expression of five abnormally methylated genes in AD and control groups and found that only *FBLN1* was significantly different in the AD and normal groups (*p* < 0.05). We also mapped the differential expression of five aberrant methylated genes in the cell cluster, with *FBLN1* being most prominently distributed in endothelial cells, with a higher expression of *FBLN1* endothelial cells in AD than in normal tissues.

We further mapped the differential expression of the 10 abnormally methylated genes across cell clusters, finding that *FBLN1* was most significantly distributed within cells, particularly endothelial cells, with higher expression in AD tissues than in normal tissues. This drew our attention because FBLN1 is an important ECM protein involved in various cellular functions such as adhesion, migration, proliferation, differentiation, and signal transduction [22]. Abnormal *FBLN1* expression has been found in various diseases, including cancer, cardiovascular diseases, and diabetes [22,27,28,29]. However, there are few studies on the role of *FBLN1* in AD. We hypothesize that *FBLN1* may participate in AD pathology by affecting endothelial cell function. Endothelial cells are the main components of the blood–brain barrier, maintaining brain homeostasis and protecting neurons from external damage [30]. In AD, the integrity and selectivity of the blood–brain barrier are compromised, leading to the infiltration of Aβ and other harmful substances and impairing the clearance of nutrients and metabolic waste [31,32]. Additionally, endothelial cells secrete various neurotrophic factors such as nerve growth factor (NGF), brain-derived neurotrophic factor (BDNF), and vascular endothelial growth factor (VEGF), which are crucial for neuronal survival and function [33,34]. In AD, the levels of these neurotrophic factors decrease, leading to neurodegenerative changes [35,36]. Therefore, we speculate that *FBLN1* may be involved in AD pathology by affecting endothelial cell function.

Finally, GO analysis revealed that *FBLN1* is mainly associated with pathways such as focal adhesion, aldosterone synthesis and secretion, and thyroid hormone synthesis. In the KEGG analysis, *FBLN1* was primarily associated with pathways such as collagen binding, small GTPase binding, and actin binding. A hallmark analysis revealed that *FBLN1* is mainly related to pathways such as UV-response-dn, protein secretion, mitotic spindle, and hypoxia. *FBLN1* may regulate the occurrence and development of AD through these pathways.

To verify our hypothesis, we conducted a series of experiments, including RT-PCR and Western blotting, to detect changes in the methylation status and protein levels of the *FBLN1* gene. We found that the exon region of the *FBLN1* gene exhibited significant hypomethylation during the mid-stage of AD. We used RT-PCR and Western blotting to further analyze the mRNA and protein expression of *FBLN1* in the hippocampal tissues of DKO and DTG mice to understand the impact of *FBLN1* gene methylation modifications on its transcriptional and translational levels in the hippocampus. The results showed no significant difference in *FBLN1* expression between the experimental and control groups in early-stage AD hippocampal tissues, consistent with RRBS results. However, in mid-stage AD hippocampal tissues, the mRNA and protein expression of this gene significantly increased, consistent with previous findings. This indicates that FBLN1 protein expression increases with age and is associated with AD pathology.

FBLN1 is a multifunctional extracellular matrix protein produced by neurons, involved in various cellular functions such as growth, adhesion, and migration [37,38]. *FBLN1* has also been implicated in the development of various cancers. Overexpressed variants of *FBLN1* play a crucial role in estrogen-induced carcinogenesis in ovarian and breast cancers. Additionally, high methylation of the *FBLN1* promoter is associated with the progression of hepatocellular carcinoma, squamous cell carcinoma, and skin cancer [39,40,41,42]. DNA methylation has been found in the promoters and genomes of genes related to AD patients [43]. Specifically, 5-methylcytosine can alter the structure of local chromatin, thereby affecting gene transcription and expression [44].

Previous studies have confirmed the association of *FBLN1* with neurodegenerative diseases [45], but there are no reports on the methylation modifications of *FBLN1* in hippocampal tissue. Interestingly, we observed a discrepancy in our study. Previous research suggested that BDNF is hypermethylated in the peripheral blood of AD patients [46], but another study found no significant difference [47]. This discrepancy may be due to differences in experimental platforms, methods, individual variations, and other confounding factors. In our experiments, *FBLN1* showed no significant methylation differences in the early stages but exhibited significant hypomethylation in the mid-stage, which requires further confirmation.

Additionally, Shen et al. reported findings on *FBLN1* expression in AD consistent with our results [48]. Generally, aberrant gene methylation can alter chromatin structure, impacting gene transcription and expression, thereby contributing to disease development [49]. These findings suggest that *FBLN1* may influence AD progression not through Aβ protein deposition but by upregulating RNA and protein expression. Studies have also reported that *FBLN1* can inhibit the proliferative effects of sAPPα fragments on neural stem cells in vivo [50]. Under physiological conditions, the C83 fragment produced by APP cleavage can generate the APP intracellular domain (AICD) through γ-secretase hydrolysis, which may contribute to AD pathology mediated by tau protein [51]. This indicates that *FBLN1* may mediate neuronal loss and be related to the development and progression of AD, although its specific mechanisms require further investigation.

The limitations of this study include the validation and analysis of only a single aberrantly methylated gene. We did not further investigate other genes identified through our screening, nor did we explore the upstream- and downstream-signaling pathways of the aberrantly methylated genes. Additionally, we did not experimentally validate the functions of these genes, assess their expression and roles in human AD patients, or evaluate the impact of interventions targeting these genes on AD progression. While the DKO and DTG mouse models used in this study are ideal for age-dependent neurodegenerative disease and AD, respectively, they cannot fully replicate all AD pathologies, let alone the epigenetic features and potential dynamic changes in AD patients. These limitations likely contribute to discrepancies between preclinical and clinical findings. Therefore, it is crucial to investigate *FBLN1* gene-methylation changes and their impact on AD pathology in human brain tissue.

Furthermore, the sample size in this experiment was limited. Although we attempted to increase the number of experimental animals, it is still challenging to completely eliminate the influence of individual differences and human factors in some mice. Thus, our experimental results require corroboration with larger sample sizes and additional experimental data. Future research should address these issues to provide more evidence and insights into the mechanisms, prevention, diagnosis, and targeted treatment of AD.

## 4. Materials and Methods

### 4.1. Experimental Animals

Studies have shown that the prevalence of AD is higher in females than in males due to hormonal influences [37]. Therefore, female AD animal model mice were used in this experiment. The experimental DKO mice were provided by Professor Ya-Ping Tang from Louisiana State University, USA. These mice were generated using the Cre/Loxp system, where the Cre gene was specifically expressed in the forebrain of B6CBAF1 background mice, leading to the conditional knockout of the Presenilin-1 (PS1) gene in the forebrain. These mice were then crossed with Presenilin-2 (PS2)-knockout heterozygous mice to obtain the DKO mice with the genotype PS1 F/F, PS2 −/−, Cre +/−.

**Figure 10:**
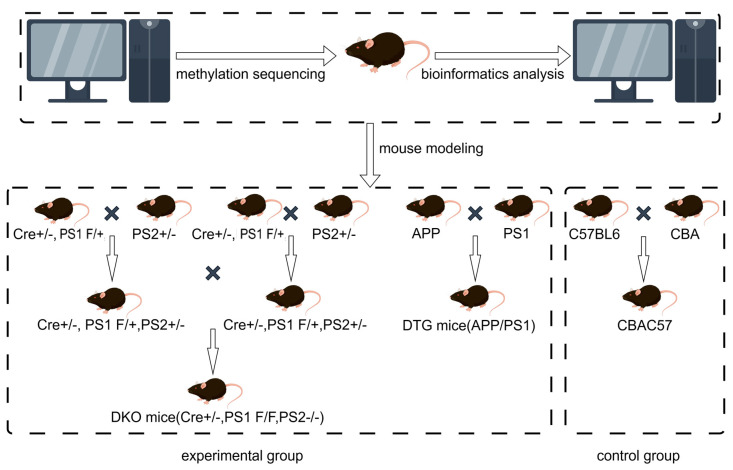
Modeling process of DKO, DTG mice.

All DKO mice used in this study were offspring that had been bred for over 10 generations and were verified to meet the genotype requirements. The DTG mice were purchased from Beijing HFK Bioscience Co., Ltd., Beijing, China, and have a C57BL/6J genetic background. They were created by crossing two single-transgenic mouse lines: PrP-hAPPK595N/M596L and PrP-hPS1dE9. Additionally, wild-type CBAC57 mice, used as controls, were purchased from Shanghai SIPPR-BK Laboratory Animal Co., Ltd., Shanghai, China. These wild-type mice were hybrids of C57BL/6 and CBA mice, with the genotype PS1 +/+, PS2 +/+, Cre −/− (Figure 10).

### 4.2. Experimental Grouping

Early Stage (7 months old): 18 female DKO mice and 18 female CBAC57 mice were divided into experimental and control groups, respectively. The experimental group consisted of nine female DKO mice and the control group consisted of nine female CBAC57 mice, used for detecting FBLN1 protein levels. Similarly, nine female DKO mice and nine female CBAC57 mice were used for detecting *FBLN1* mRNA levels.

Mid-Stage (12 months old): A total of 18 female DKO mice, 18 female DTG mice, and 18 female CBAC57 mice were used. For protein-level detection, nine female DKO mice, nine female DTG mice, and nine female CBAC57 mice were used. For mRNA-level detection, nine female DKO mice, nine female DTG mice, and nine female CBAC57 mice were used [8]. Wild-type CBAC57 mice were used as controls.

### 4.3. Collection of Tail Tissue from Offspring Mice

Approximately 40 days after birth, tail tissue from the offspring mice in the experimental groups was collected. Under sterile conditions, the offspring mice were anesthetized. Surgical ligation was performed about 0.6 cm from the tip of the tail, and approximately 0.5 cm of tail tissue was cut using a scalpel. An ophthalmic scissor was used to mark the mouse’s ear. The tail tissue was placed in a sterilized and dry 1.5 mL centrifuge tube. The wound was disinfected twice with 75% alcohol. If necessary, additional ligation was performed to stop the bleeding. Once the mice recovered from anesthesia, they were returned to their cages.

### 4.4. Collection of Hippocampal Tissue

The mice were anesthetized with ether and decapitated. The skin and skull were removed to expose the entire brain. The hippocampus was then isolated, quickly frozen in liquid nitrogen for 30 min, and stored at −80 °C in a freezer [8].

### 4.5. Methods

#### 4.5.1. DNA Extraction from Mice

DNA extraction reagents were purchased from Tiangen Biotech Co., Ltd., Beijing, China (catalog number DP304). Mouse DNA was extracted using these reagents. The extracted DNA was amplified using standard PCR. The PCR primers used are listed in Table S1.

#### 4.5.2. Agarose Gel Electrophoresis Detection

(1) Gel Preparation: A 1.2% agarose gel was prepared for electrophoresis. (2) Electrophoresis: We loaded 4 µL of DNA Marker and 5 µL of the PCR amplification products from each sample, including positive and negative controls, into the wells of the agarose gel. Electrophoresis was performed at a constant voltage of 150 V, with a current of 125 A and power of 180 W, for a duration of 22 min. (3) Imaging: Following electrophoresis, the gel was transferred to an automated UV transilluminator for visualization. Images of the gel were captured and saved. Offspring mice were genotyped as DKO and DTG based on the expected DNA fragment sizes and their comparison to the positive control.

#### 4.5.3. Reduced Representation Bisulfite Sequencing (RRBS)

In this experiment, RRBS sequencing was conducted by Hangzhou Lianchuan BioTech Co., Ltd., Hangzhou, China. The construction of the sequencing library was performed as described by Brant et al. [52]. The principle involved treating genomic DNA with the restriction enzyme MspI to enrich for the promoter and CpG island regions, followed by bisulfite conversion, which converted unmethylated cytosines to uracil. After PCR amplification, all uracils were converted to thymine. The PCR products were then sequenced. The sequencing results were analyzed by Bismark (v 0.7.11) and other software, and a series of genes with abnormal methylation changes were finally selected. Genes with *p* < 0.05 were selected. Finally, the top 25 genes with more significant *p*-values associated with a poor prognosis of Alzheimer’s disease (AD) were included for further analysis.

#### 4.5.4. PPI (Protein–Protein Interaction)

The core network of protein–protein interactions (PPICN) refers to the integration of biochemical, signal transduction, and genetic networks, associating compounds with disease-related protein molecules. We imported the drug-disease crossover targets into the STRING database (https://cn.string-db.org/, accessed on 15 May 2024), setting the species to Homo sapiens. The data were saved in tab-separated values (TSV) format. The TSV file was imported into Cytoscape 3.7.1 for network analysis. Node sizes were adjusted based on the degree values from the analysis results. To further quantify the importance of these genes in the PPI network, we used the MCODE and cytoHubba tools to quantitatively rank the core genes [53].

#### 4.5.5. Single-Cell Sequencing (ScRNA-Seq)

Transcriptome data of AD and normal tissues (GSE161045) were obtained from the Gene-Expression Omnibus database (GEO). The ScRNA-seq data were processed using the Seurat V4.0 R package. First, the “PercentFeatureSet” function was used to calculate the proportion of mitochondrial genes, selecting cells with more than 200 genes but fewer than 5000 genes, and fewer than 2000 mitochondrial genes, for further analysis. Then, the ScRNA-seq data were normalized using the “NormalizeData” method, and five highly variable genes (HVG) were enriched using the “FindVariableGenes” function. Next, the “FindIntegrationAnchors” and “IntegrateData” functions were used to merge and regress the ScRNA-seq data from AD and normal tissues to account for batch effects. The “FindNeighbors” and “FindClusters” functions were then performed to identify cell clusters (resolution = 8.0). Finally, these cell clusters were visualized using the UMAP and tSNE methods and annotated based on previously reported marker genes.

#### 4.5.6. Extraction of DNA from Hippocampal Tissue

We removed 200 mg of frozen brain tissue, placed it in a 2 mL centrifuge tube, and added 1.4 mL of buffer ASL. We allowed it to thaw completely, then vortexed for 1 min to mix thoroughly. The mixture was incubated at 70 °C for 5 min with continuous shaking for 15 s, then we let it stand and centrifuged at high speed. We retained the supernatant and transferred it to an EP centrifuge tube. We added 100 µL of AB clearing agent to remove impurities, vortexed for 1 min to mix thoroughly, closed the cap, let it stand, and centrifuged at high speed. Then, we transferred all the supernatant to a new EP centrifuge tube and centrifuged at high speed again. Next, we transferred 210 µL of the supernatant to a new, clean EP centrifuge tube, added 20 µL of proteinase K solution, mixed it well, added 200 µL of CB, and again mixed well. This mixture was incubated at 70 °C for 10 min. After incubation, we added 100 µL of isopropanol and mixed well. Then, we added the solution to adsorption column AC, centrifuged it at high speed, and discarded the waste liquid. We added 500 µL of IR inhibitor removal solution to AC, centrifuged it at high speed, and discarded the waste liquid again. Next, we added 600 µL of WB wash solution to AC slowly and evenly, centrifuged it, and discarded the waste liquid. We repeated the wash cycle once, then placed AC in a clean recovery tube and centrifuged it at high speed to remove the wash solution. We discarded the recovery tube, placed AC in a new recovery tube, and eluted it with 50 µL of EB elution buffer. Finally, we centrifuged the mixture at high speed, and the resulting solution was the DNA solution.

#### 4.5.7. DNA Quality Identification and Integrity Assessment

In this experiment, a micro-nucleic acid protein detector (Analytik Jena, Beijing, China) was used to measure the DNA OD value, using the A260/A280 ratio. Additionally, 1% agarose gel electrophoresis was used to assess DNA integrity.

#### 4.5.8. Bisulfite Treatment

First, we took 45 µL of DNA solution and added 5 µL of pre-prepared Solution A. We let it stand for 15 min with the cap closed, then added 500 µL of Solution B and mixed well. This was stored for 8–16 h, then placed in an ice bath for 10 min. We added the universal sol solution, mixed it well, and centrifuged it at high speed. Next, we added 0.7 mL of universal wash solution to the column and centrifuged it at high speed (12,000 rpm, 30 s). Then, we added 50 µL of Solution A diluent, let it stand at room temperature for 2 min, and centrifuged it at high speed. Next, we added 0.7 mL of universal sol solution, mixed it well, applied it to the column, and centrifuged it at high speed. Finally, we added 50 µL of DNA elution solution, let it stand at room temperature for 2 min, and centrifuged it at high speed (12,000 rpm, 30 s) to obtain bisulfite-modified DNA.

#### 4.5.9. PCR Amplification and Product Recovery

This experiment amplified fragments containing the promoter region and all exon sequences of the target gene. According to the kit instructions, the gel-cutting method was used to recover and purify the amplified target products.

#### 4.5.10. Real-Time Quantitative Polymerase Chain Reaction (RT-PCR/QPCR)

RNA Extraction

Hippocampal tissue was ground in liquid nitrogen, followed by the addition of RNAiso Plus (10× tissue weight). The mixture was transferred to an RNase-free tube, left to stand for 5 min at room temperature, and centrifuged at 4 °C, 12,000 rpm for 5 min. The supernatant was mixed with 200 µL of chloroform, left to stand for 5 min, then centrifuged again. The new supernatant was combined with 100 µL of isopropanol, mixed, left to stand for 10 min, and centrifuged at 4 °C, 12,000 rpm for 10 min. The resulting pellet was washed with 75% ethanol, centrifuged, and air-dried on ice for 50 min. Finally, the pellet was dissolved in 20 µL of DEPC-treated water, yielding the total RNA solution.

RNA Concentration and Purity Detection

First, we cleaned the UV spectrophotometer with RNase-free water and set it to zero. We measured the RNA concentration and purity by taking 1–2 µL of the solution from the EP tube, repeating the measurement three times. We recorded the average value as the final concentration. The OD value should be between 1.8 and 2.2, with a 28S/18S ratio between 1.8 and 2.0.

cDNA Template Preparation (Reverse Transcription)

Based on the detected RNA concentration of each sample, we diluted all samples to a final RNA concentration of 500 ng/µL using RNase-free water. We used the RT reagent Kit with gDNA Eraser (TaKaRa, Beijing, China). The reaction conditions were as follows: 37 °C for 15 min, 85 °C for 5 s, and 4 °C for 5 min.

RT-PCR

Primers for the *FBLN1* gene and the reference gene GAPDH were designed and synthesized by Shanghai Sangon Biotech Co., Ltd., Shanghai, China. We prepared the PCR reaction system according to standard protocols, with three replicates for each sample. The reaction conditions were as follows: 40 cycles at 95 °C for 35 s, 60 °C for 30 s, and 95 °C for 30 s.

Total Protein Extraction

We obtained hippocampal tissue from mice (approximately 8–12 mg per mouse) and ground it into a fine powder in liquid nitrogen. We added lysis buffer (PMSF: RIPA = 1:100, 10 µL/mg tissue) and lysed on ice for 35 min. Then, we sonicated the mixture for 5 s and centrifuged it. We retained the supernatant as the total protein solution.

Protein Concentration Measurement

We measured the protein concentration using the Bioepitope BCA protein assay kit (Beyotime, Shanghai, China) according to the manufacturer’s instructions. We mixed the protein sample with 5×SDS-PAGE sample buffer at a 1:4 ratio and denatured the solution in a water bath.

Western Blot Detection
Gel Preparation and Sample Loading

First, we prepared a 10% resolving gel and a 5% stacking gel by mixing Solution A and Solution B in a 1:1 ratio and adding the polymerization accelerator. We poured the resolving gel into the gel-casting glass plate, flattened it with ethanol, and allowed it to solidify. Next, we layered the stacking gel on top of the resolving gel and inserted the sample comb.

Electrophoresis

We set up the electrophoresis apparatus properly. We ran the stacking gel at a constant voltage of 80 V for 30 min.

Electrotransfer

We activated the PVDF membrane with methanol, soaked the filter paper in pre-cooled transfer buffer, and cut gel strips corresponding to the molecular weights of FBLN1 and β-actin proteins. Next, we assembled the transfer sandwich—sponge, filter paper, gel strip, PVDF membrane, filter paper, and sponge—ensuring no air bubbles. Then, we transferred the FBLN1 protein at a constant current of 200 mA on ice for 100 min and β-actin protein for 70 min.

Blocking and Antibody Incubation

We blocked the PVDF membrane with 5% non-fat milk, then incubated it with primary antibodies overnight at 4 °C. We washed the membrane with TBST and incubated it with secondary antibodies for 1 h.

Statistical Methods

Statistical analysis was performed using SPSS 26.0 software, and graphs were created with GraphPad Prism 8.0. Gene expression at the protein and transcription levels was analyzed using the one-sample *t*-test. The measurement data were expressed by (*x* ± *s*)*. p* < 0.05 was considered statistically significant.

## 5. Conclusions

In summary, after the 25 methylated sequenced genes were screened, five core genes were screened again by PPI for further single-cell sequencing analysis. It was found that *FBLN1* was significantly expressed in single-cell sequencing, and its expression was higher in the AD group than in the control group. Experimental validation revealed that the *FBLN1* gene expression in AD mice and normal group mice was the same as in the single-cell sequencing analysis, suggesting that hypomethylation of the *FBLN1* gene may be involved in the mid-term pathological process of AD by promoting gene expression. These results suggest that there is some non-Aβ deposition mechanism, or multiple factors may be involved in the epigenetic regulation of AD process in age-dependent neurodegenerative lesions in conjunction with Aβ deposition. This experiment provides a theoretical basis for the study of the epigenetic and non-Aβ deposition mechanisms of AD, as well as the targeted diagnosis, treatment, and prognosis of AD genes.

## Data Availability

The datasets used and/or analyzed during the current study are available from the corresponding author upon reasonable request.

## Supplementary Materials

The following supporting information can be downloaded at: https://www.mdpi.com/article/10.3390/ijms25169036/s1.
