# Supplementary material for: A Bioinformatics-Based Study on Methylation Alterations of the FBLN1 Gene in Hippocampal Tissue of Alzheimer’s Disease Model DKO and DTG Mice
Source: Int J Mol Sci. 2024 Aug 20;25(16):9036. doi: 10.3390/ijms25169036 (PMC11354892; doi:10.3390/ijms25169036)
Supplement: Supplementary file 1 [file ijms-25-09036-s001.zip › ijms-3086708-supplementary.pdf]

Table S1 The primer sequences of PCR

Gene Name	Primer sequence (5'- 3')	Target band size
Cre - Forward primer	AGATGTTCGCGATTATC	490bp
Cre - Reverse primer	AGCTACACCAGAGACGG	
PS1 - Forward primer[a]	CAGACATTAGCACTGTCTGTAAGG	703bp
PS1 - Reverse primer[a]	GTTCCTAAACCTCTAAACTTCCATGA	
PS2 - EX6 primer	AAGTATCGATGCTACAAGGTGAGG	450bp
PS2 - Re primer	CCCACATGATAAAAGGAGAGC	
PS2 - PGK-Neo-PA primer	GCCTGAACAACGAGATCAGCA	
APP - Forward primer	GACTGACCACTCGACCAGGTTCTG	350bp
APP - Reverse primer	CTTGTAAGTTGGATTCTCATATCCG	
PS1 - Forward primer[b]	AATAGAGAACGGCAGGAGCA	608bp
PS1 - Reverse primer[b]	GCCATGAGGGCACTAATCAT	
internal reference -Forward primer	CTAGGCCACAGAATTGAAAGATCT	324bp
internal reference -Reverse primer	GTAGGTGGAAATTCTAGCATCATCC	

Table S2 The partial results of DMC

Chromosome	Initiation site	Termination site	*P value*	Annotated originals	Gene Name
chr5	135789302	135789625	0.019802	intron	Mdh2
chr5	37118779	37118908	4.30E-09	exon	Jakmip1
chr5	92955261	92955412	4.30E-09	exon	Shroom3
chr1	38797464	38798664	0.023662	promoter	BC096025
chr11	114710187	114711759	0.028618	intron	Ttyh2
chr16	59440476	59440629	3.12E-08	exon	Gabrr3
chr19	40608257	40608691	0.035139	intron	Tctn3
chr5	5682534	5683056	0.009781	exon	Steap2
chr8	25698328	25699028	0.020256	promoter	Whsc1l1
chr5	122745132	122746332	0.002524	promoter	Camkk2
chr14	70153627	70153792	3.12E-08	exon	Ccar2
chr5	38501653	38502352	0.022942	promoter	Slc2a9
chr2	153891160	153891279	3.12E-08	intron	Bpifb2
chr3	90021239	90021517	0.019802	exon	Ubap2l
chr10	42185717	42186917	0.014298	promoter	AB335951
chr4	127544101	127544900	0.049256	promoter	AK039749
chr11	107672529	107673046	0.048682	exon	Helz
chr7	133909094	133909203	3.12E-08	exon	Adam12
chr17	32906133	32906288	3.12E-08	intron	Cyp4f14
chr13	24326404	24327203	0.034164	promoter	Cmah
chr7	68201348	68201901	0.015385	intron	Igf1r
chr9	108912419	108913618	0.044463	promoter	Tmem89

Table S3 The results of genes with different DMR

Chromosome	Initiation site	Termination site	*P value*	Annotated originals	Gene Name
chr2	155064078	155064153	3.12E-08	intron	AHCY
chr8	11241122	11241209	3.12E-08	exon	COL4A1
chr5	24372634	24372739	3.12E-08	exon	NOS3
chr1	91432808	91432893	1.11E-16	exon	PER2
chr13	11741854	11742081	1.11E-16	exon	RYR2
chr7	80283980	80284077	3.12E-08	exon	VPS33B
chr15	85244183	85244307	0.02	exon	FBLN1
chr1	193343374	193343879	0.011499	exon	LAMB3
chr5	134732533	134732563	0.03742	exon	ELN
chr7	102266824	102267623	0.015982	promoter	STIM1
chr8	22059575	22060274	0.026382	promoter	Atp7b
chr16	36561635	36562334	0.031065	promoter	Casr
chr7	68173231	68173376	0.025378	exon	Igf1r
chr11	108941903	108942044	0.038039	exon	Axin2
chr10	53629632	53630331	0.023083	promoter	Mcm9
chr1	21961181	21961943	0.001921	exon	Kcnq5
chr5	140612760	140612846	0.013238	exon	Lfng
chr16	16358076	16358775	0.022643	promoter	Dnm1l
chr19	29437230	29437536	0.002564	exon	Pdcd1lg2
chr9	36726159	36726858	0.006341	promoter	Chek1
chr7	28761937	28762025	3.12E-08	intron	Nfkbib
chr7	30574239	30574324	0.024858	intron	Kmt2b
chr8	70503494	70503596	0.03504	intron	Crlf1

chr2	166870210	166870383	0.039744	exon	Arfgef2
chr15	85901200	85901438	0.031561	intron	Celsr1

Table S4: MCODE and cytoHubba analysis data for the top 4 proteins

name	Closeness	Degree	Eigenvector	Information	LAC	MCODE::Clusters (1)	MCODE::Node Status (1)	MCODE::Score (1)	Betweenness	Subgragh
COL4A1	0.189655172	4	0.527810454	1.850899935	1	Cluster 1	Clustered	1.666666667	14	5.506014824
ELN	0.186440678	4	0.513366163	1.850899935	1	Cluster 1	Clustered	1.666666667	14	5.426658154
NOS3	0.186440678	3	0.426967233	1.718376994	0.666666667	Cluster 1	Clustered	2	10	4.084474087
FBLN1	0.177419355	2	0.358657777	1.535181046	1	Cluster 1	Seed	2	0	3.202656031

Table S5 Methylation status of *FBLN1* gene by RRBS

Chromosome	Gene Name	Initiation site	Termination site	Annotated originals	Methylation Status	*P* *value*	Age (months)
chr15	*FBLN1*	85244183	85244307	exon	hypomethylation	0.002	12
chr15	*FBLN1*	85285040	85286295	exon	hypomethylation	0.190	7

Table S6 The mRNA expression of *FBLN1* gene in hippocampus of DKO mice, DTG mice and CBAC57 mice($\bar{x}$ ± s)

AD Disease Progression	divide into groups	Relative Expression of *FBLN1* Gene
Early Stage	DKO (7 months)	1.372 ± 0.179
	CBAC57 (7 months)	1.024 ± 0.255
Mid Stage	DKO (12 months)	2.769 ± 0.603##
	CBAC57 (12 months)	1.008± 0.157
Mid Stage	DTG (12 months)	1.959 ± 0.408*
	CBAC57 (12 months)	1.013± 0.192

Note:#compared with 12-month-old CBAC57, ##*P* < 0.01 ; *compared with 12-month-old CBAC57, **P* < 0.05.

Table S7 The protein expression of *FBLN1* gene in hippocampus of DKO mice, DTG mice and CBAC57 mice($\bar{x} \pm s$)

AD Disease Progression	divide into groups	Relative Expression of *FBLN1* Gene
Early Stage	DKO (7 months)	1.112 ± 0.034
	CBAC57 (7 months)	1.000 ± 0.089
Mid Stage	DKO (12 months)	1.563 ± 0.052##
	CBAC57 (12 months)	1.000 ± 0.116
Mid Stage	DTG (12 months)	1.626 ± 0.085**
	CBAC57 (12 months)	1.000 ± 0.114

Note: # Compared to 12-month-old CBAC57, ##$P < 0.01$; * Compared to 12-month-old CBAC57, **$P < 0.01$.
